# Population Trend and Elasticities of Vital Rates for Steller Sea Lions (*Eumetopias jubatus*) in the Eastern Gulf of Alaska: A New Life-History Table Analysis

**DOI:** 10.1371/journal.pone.0140982

**Published:** 2015-10-21

**Authors:** John M. Maniscalco, Alan M. Springer, Milo D. Adkison, Pamela Parker

**Affiliations:** 1 Science Department, Alaska SeaLife Center, Seward, Alaska, United States of America; 2 Institute of Marine Science, University of Alaska Fairbanks, Fairbanks, Alaska, United States of America; University of Missouri Kansas City, UNITED STATES

## Abstract

Steller sea lion (*Eumetopias jubatus*) numbers are beginning to recover across most of the western distinct population segment following catastrophic declines that began in the 1970s and ended around the turn of the century. This study makes use of contemporary vital rate estimates from a trend-site rookery in the eastern Gulf of Alaska (a sub-region of the western population) in a matrix population model to estimate the trend and strength of the recovery across this region between 2003 and 2013. The modeled population trend was projected into the future based on observed variation in vital rates and a prospective elasticity analysis was conducted to determine future trends and which vital rates pose the greatest threats to recovery. The modeled population grew at a mean rate of 3.5% per yr between 2003 and 2013 and was correlated with census count data from the local rookery and throughout the eastern Gulf of Alaska. If recent vital rate estimates continue with little change, the eastern Gulf of Alaska population could be fully recovered to pre-decline levels within 23 years. With density dependent growth, the population would need another 45 years to fully recover. Elasticity analysis showed that, as expected, population growth rate (λ) was most sensitive to changes in adult survival, less sensitive to changes in juvenile survival, and least sensitive to changes in fecundity. A population decline could be expected with only a 6% decrease in adult survival, whereas a 32% decrease in fecundity would be necessary to bring about a population decline. These results have important implications for population management and suggest current research priorities should be shifted to a greater emphasis on survival rates and causes of mortality.

## Introduction

Long-term studies of vital rates among wild animals are invaluable for understanding population dynamics [1], and changes in population trends can be well explained by life-history modeling [2]. Leslie [3,4] laid the groundwork for modeling population dynamics with matrices based on age-structured vital rates. This theoretical work has since been widely expanded upon and aids our understanding of many aspects of declining, stable, and increasing populations [5]. Furthermore, life table matrices are the basis for exploring the effects of changing vital rates on population trends, providing a highly informative tool for identifying life stages that should be targeted for species management [6,7].

Steller sea lions (*Eumetopias jubatus*) have received much research and management attention since experiencing a steep decline in numbers between the 1970s and the 1990s. This decline prompted the 1997 listing of the western distinct population segment (WDPS) as endangered under the Endangered Species Act of the United States (Federal Register 62:30772–30773). The declines were primarily attributed to decreased juvenile survival, an increase in mean adult female age, and a slight decrease in reproductive rates [8]. To date, all published life table modeling studies of Steller sea lion populations have relied heavily on observed age structure from surveys or collected animals [8–10]. However, such estimates can be highly imprecise because they do not account for, or poorly account for, detection probabilities, sampling variation, and biological process variation [11,12]. Estimates of Steller sea lion vital rates based on longitudinal studies, which do account for detection probabilities and some sources of sampling and process variation, are now widely available [13–17]. These data can be modeled in life history matrices to understand population trends and potential threats to population recovery that are, in some ways, more precise than using snapshot counts of population age structure [1,12].

Steller sea lions are pinnipeds and considered to be a *K*-selected species, or at the slow end of the life-history continuum [18]. Females give birth to one offspring per year but not necessarily every year, have long lactation periods (9 mo to 4 yr), and do not reach sexual maturity until 4.5 yrs of age on average [19,20]. Leading hypotheses for their population decline include predation by killer whales (*Orcinus orca*), reductions in the availability of important prey items, or a combination of those factors in addition to conflicts with commercial fisheries [21–23]. By the early 2000s, much of the WDPS had stopped declining, and numbers have begun to increase in some areas [24]. The upswing in numbers may be attributed to improved juvenile survival and high reproductive rates [14,16,25]. However, given their relative position at the slow end of the life-history continuum, recovery of Steller sea lions, like other pinniped populations, can take decades [26,27].

Substantial variation in vital rates that directly influence population trends can be expected with natural environmental variation and climate change [2,28,29]. Environmental perturbations may cause changes in life history traits that covary positively with each other; e.g., a reduction in food supply may inhibit both fecundity and survival rates [2]. Yet, vital rates that have the greatest influence on population trends tend to be more stable, as explained by the demographic buffering hypothesis [30,31]. Among long-lived marine animals, stability in adult survival generally buffers against greater temporal variance in fecundity and juvenile survival [29,32]. Steller sea lions may have an additional buffering mechanism brought about by vital rates that covary negatively. That is, mature females that skip or abort a pregnancy in one year, tend to nurse their dependent offspring for an additional year [19]. Such behavior greatly increases survival probability of juveniles between 1 yr and 2 yrs of age [16]. Therefore, reductions in fecundity can be offset to some degree by an increase in juvenile survival. In this manner, Steller sea lion populations may continue to increase even during periods of reduced reproduction.

The purpose of this study was to apply current mark-recapture-based vital rate estimates of Steller sea lions to a population matrix model to assess the rate of the population recovery in the eastern Gulf of Alaska. This population is not closed [33,34] and therefore, population trends determined from life tables may not agree with trends estimated from survey counts. Thus, the work we present here compares the modeled population trends with survey counts over the years 2003–2013 to determine the level of correlation, and forecasts future trends based on observed variation in vital rates and density dependent logistic growth to estimate the strength of the recovery that is possible regardless of immigration or emigration. Additionally, a prospective elasticity analysis [35] is presented to determine the greatest demographic threats to population recovery. Elasticity analysis is a simple and powerful tool used to identify how much influence each vital rate has on the population trend, independent of variation in vital rates, helping to pinpoint the most appropriate life history stages to be targeted for management objectives [6].

## Methods

This research was approved by Alaska SeaLife Center Institutional Animal Care and Use Committee Protocol No. R10-03-01 and National Marine Fisheries Service Permit Nos. 14324 and 18438 for research on endangered Steller sea lions. The Chiswell Island group is part of the U. S. Fish and Wildlife Service National Maritime National Wildlife Refuge. Research was conducted on refuge lands under Right-of-Way Permit No. M-344-AM and Special Use Permit No. 74500-10-001 and earlier versions.

**Fig 1:**
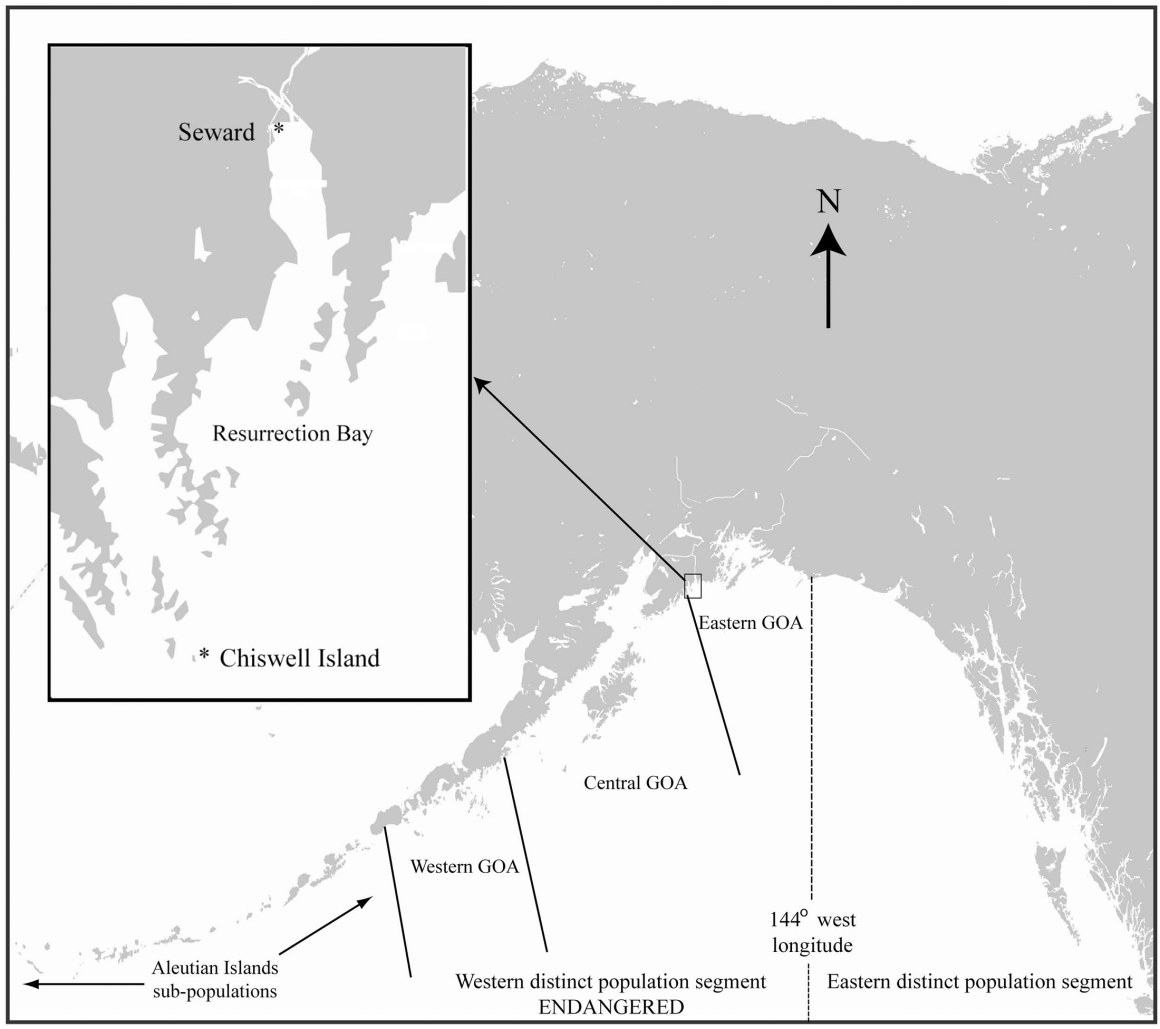
Map of the study area (inset) in the eastern Gulf of Alaska. The division of Steller sea lion populations into the eastern and western distinct population segments at 144° west longitude, as well as other subpopulations is noted.

The focal area of this study was the Steller sea lion rookery on Chiswell Island (59° 36.13' N, 149° 34.05' W) in the eastern Gulf of Alaska (EGOA), part of the endangered WDPS (Fig 1), observed over the years 2003 to 2013. The population decline at the Chiswell rookery was similar to that of other rookeries in the Gulf of Alaska; abundance fell by 90% from 1,106 adults in 1976 [36] to approximately 90 adults and 50–80 pups in the 2000s [37].

Steller sea lions were monitored from at least 6:00 AM to 10:00 PM using 4 to 6 remotely operated video cameras placed on the island (see [37] for details). Complete census counts of all sea lions by age class (adult males, adult females, juveniles 1 yr to 4 yrs old, and pups 0 to 1 yr old) on the rookery were conducted at approximately 11:00 AM and 7:00 PM between 20 June and 10 July during all years of the study. This date range corresponds to range-wide flight surveys for population counts conducted by the National Marine Fisheries Service [33], thus allowing comparisons with broader regional data and modeled population trends.

**Fig 2:**
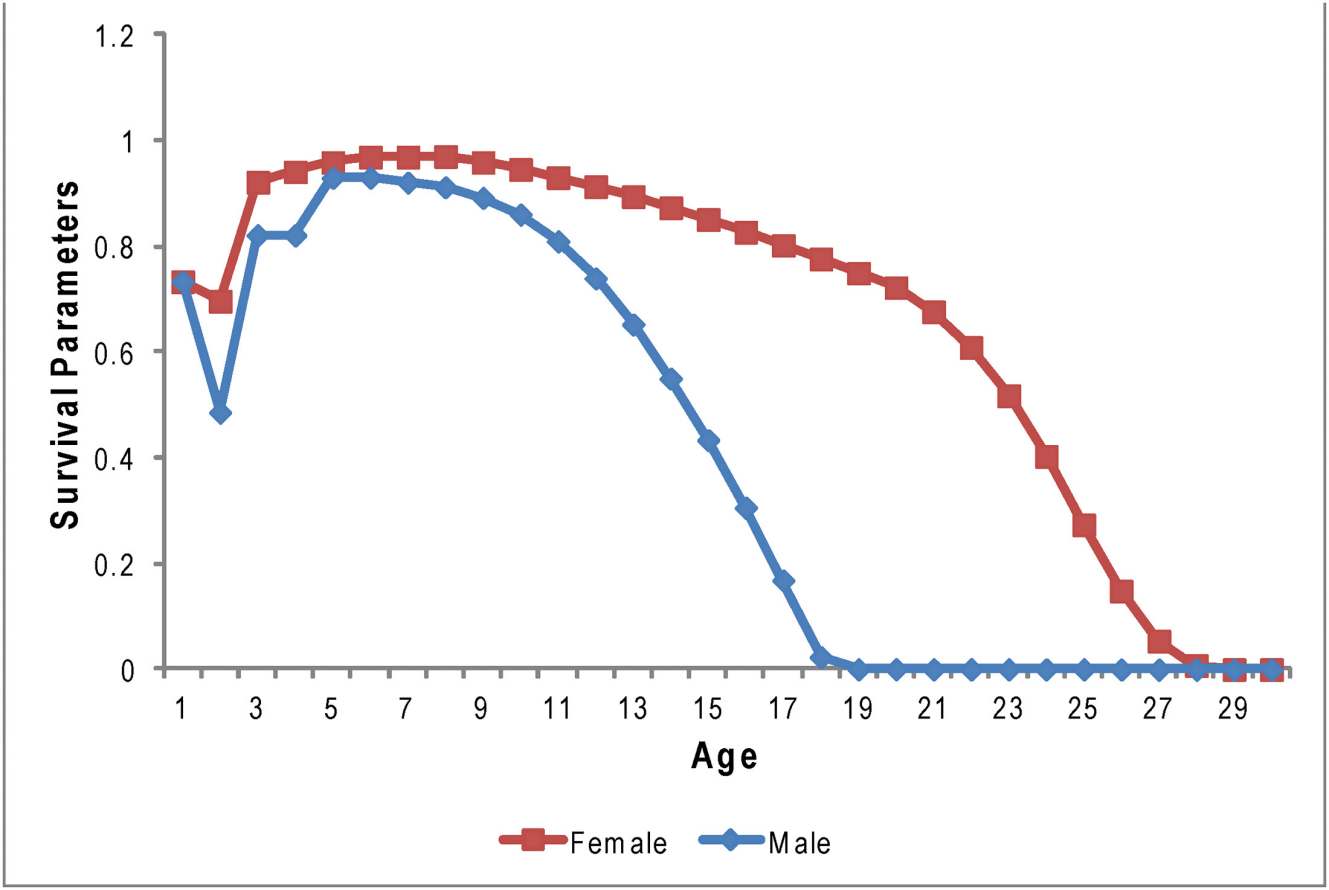
Male and female survival parameters used in the life table matrices for estimates of Steller sea lion population trends in the eastern Gulf of Alaska. Age 1 survival was specific to year, while survival to ages 2+ varied between the sexes but was the same across years based on published data (Maniscalco 2014, Maniscalco et al. 2014) and a beta distribution on age. See S1 Table for complete age-specific survival rates of males and females used in this analysis.

Fecundity (also referred to as natality) and survival rates, previously estimated for this rookery [16,17], were used as input data to a life table matrix generally following recommendations by Caswell [5] (S1 Table). The year 2002 starting population was based on pup and non-pup population counts at rookeries and haulouts in the EGOA between 2000 and 2002 by the National Marine Fisheries Service [38,39], with non-pup numbers following sex-specific age distributions based on survival probabilities outlined below. The total population of age 1+ Steller sea lion abundance for each subsequent year (2003–2013) was defined as:

$$N_{t}=\left(\;\overset{n}{\underset{i=1}{\sum }}N_{x,t-1}^{f}\cdot p_{x,t}\right)+\left(\;\overset{n}{\underset{i=1}{\sum }}N_{x,t-1}^{m}\cdot q_{x,t}\right)$$

where $N_{x,t-1}^{f}$ and $N_{x,t-1}^{m}$ are the female and male proportions of the population, respectively. Survival probabilities for females (*p*_x,t_) and males (*q*_*x*,*t*_) were specific to age (*x*) but not specific to year (*t*), except for first year survival as follows (Fig 2; S1 Table). First year survival for the years 2005–2013 was modeled identically for males and females [16] as the product of observed neonatal survival for each year [40], and a year-invariant survival from approximately 3 weeks to 1 year of age (80.1%; [16]). For years 2003 and 2004, first year survival may have been lower [14], and therefore was modeled based on sex-specific estimates by Fritz et al. [14] multiplied by neonatal survival for those years [40]. Juvenile survival from ages 1 to 4 was specific to age and sex (*p*_*x*_^*j*^ and *q*_*x*_^*j*^ for females and males respectively), but did not vary across years [16]. Adult female survival (*p*_*x*_^*a*^, ages 5+) also did not vary across years and was modeled using a beta distribution with a peak at 8 yrs of age and an average of 88.1% between 5 and 20 years of age [17], after which survival dropped off sharply. Subadult and adult male survival estimates are currently unavailable for the EGOA. Therefore, survival of males at ages 5+ (*q*_*x*_^*a*^) was modeled with a beta distribution that had a peak at age 6 and dropped off more sharply after the males reached full maturity (10–11 yrs) as found by Altukhov et al. [13] (Fig 2; S1 Table). Throughout the remainder of this document, further reference to adult survival without specification to sex refers to combined male and female adult survival.

The number of new pups joining the population each year was assumed to be equal between the sexes and defined as:

$$m_{t}=\;F_{t}\cdot \;N_{t}^{F}$$

with $N_{t}^{F}$ being the number of mature females (age 5–20) in year *t*, and *F*_*t*_ being the fecundity (natality rate) of adult females specific to years 2003–2012 [17] and based on the 10-yr running average for 2013 and future projections. Females greater than 20 years of age were considered senescent [41].

Published vital rates were randomly re-sampled 1000 times within error estimates to obtain 95% bootstrap confidence intervals for the modeled population trend. Population estimates from the life table were subsequently tested for correlations with maximum census counts of age 1+ sea lions from each breeding season at the Chiswell Island rookery and with counts from the broader Gulf of Alaska region across the study period. The population trend for the EGOA was then projected into the future by varying vital rate estimates stochastically based on observed variation in the fecundity and survival over the study period (S1 Table). Random selections (n = 1000) were made from the 10 values of fecundity and within confidence intervals of survival estimates [13–16], and applied to life history matrices to obtain bootstrapped estimates of the population trend with 95% confidence intervals. This analysis was conducted without density dependence because some recovering pinniped populations go through an extended period of density independent growth [42], and it was of interest to determine the earliest predictable time to full recovery of the Steller sea lion population in the study area.

The population was also projected using the logistic growth equation, assuming a stable age distribution [43]:

$$\frac{dN}{dt}=\;rN\;\left(1-\;\frac{N}{K}\right)$$

where the intrinsic maximum rate of increase (*r*) over the study period was defined by the dominant eigenvalue of λ (asymptotic population growth rate) with *r* = ln λ [35]. This projection assumed a carrying capacity (*K*) of 10,000 Steller sea lions, which is roughly similar to population counts in the EGOA during the late 1970s when Steller sea lion abundance was at a peak in this region [36].

Population model projections were subjected to a prospective elasticity analyses [5,35] by varying the vital rate estimates described above to ascertain the greatest potential detriments to population recovery. Ideally, these rates should be covaried based on their relative correlations to each other [30,44]. However, juvenile and adult survival did not vary significantly across the study period [16,17] and there was no correlation found between fecundity and first-year (pup) survival. Vital rates, including age at maturity, were varied proportionally and systematically to determine their effect on λ. Multiple vital rates were also varied together in various combinations to assess possible synergistic effects on population projections. An additional set of scenarios tested for an effect of negative covariation between fecundity and juvenile survival by increasing survival to age 2 as fecundity declined. Females that skip a year of pupping tend to nurse their previous offspring for an additional year, thus improving its chances of survival. Therefore, as fecundity declined, survival of female offspring to age 2 was increased proportionally between 0.642 and 0.882 based on survival estimates for females weaned at age 1 and those not weaned until age 2, and between 0.406 and 0.882 for males during that same age period [16]. This allowed for modest, but realistic improvements in juvenile survival that can offset a reduction in fecundity in this species.

## Results

Using current vital rates (S1 Table) in a life table matrix, the reconstructed Steller sea lion population increased yearly over the study period. This trend held true for pups, mature females, and total age 1+ animals, although juvenile numbers were somewhat irregular due to variation in neonatal and first year survival across the study period (Table 1). Growth in the reconstructed population resulted in a decrease in the mean age of 3 yr old and older females from 9.8 yrs in 2003 to 8.5 yrs in 2013. The population did not converge on a stable age distribution during this time period, but would be expected to do so within the next 10 yrs to 15 yrs if vital rates do not change significantly in the near future.

**Table 1:** Life table population estimates for females (F) and the total age 1+ male and female population (MF). Starting population in 2002 based on survey counts in the EGOA (DeMaster 2009, 2011).

Age (F)	2002	2003	2004	2005	2006	2007	2008	2009	2010	2011	2012	2013
**1**	158.0	171.2	260.0	322.4	303.5	295.7	294.7	289.0	331.4	308.8	345.8	319.5
**2**	110.0	110.0	119.2	181.1	224.5	211.4	205.9	205.2	201.2	230.8	215.1	240.8
**3**	101.4	101.4	101.4	109.8	166.8	206.8	194.7	189.7	189.0	185.4	212.6	198.1
**4**	95.4	95.4	95.4	95.4	103.3	156.9	194.5	183.2	178.5	177.8	174.4	200.0
**5**	137.1	91.4	91.4	91.4	91.4	99.0	150.4	186.4	175.5	171.0	170.4	167.1
**6**	132.8	132.8	88.5	88.5	88.5	88.5	95.9	145.6	180.6	170.0	165.6	165.1
**7**	128.6	128.6	128.6	85.8	85.8	85.8	85.8	92.9	141.1	175.0	164.7	160.5
**8**	124.8	124.8	124.8	124.8	83.2	83.2	83.2	83.2	90.1	136.9	169.7	159.8
**9**	119.6	119.6	119.6	119.6	119.6	79.7	79.7	79.7	79.7	86.4	131.2	162.7
**10**	113.1	113.1	113.1	113.1	113.1	113.1	75.4	75.4	75.4	75.4	81.7	124.0
**11**	105.2	105.2	105.2	105.2	105.2	105.2	105.2	70.1	70.1	70.1	70.1	76.0
**12**	96.0	96.0	96.0	96.0	96.0	96.0	96.0	96.0	64.0	64.0	64.0	64.0
**13**	85.8	85.8	85.8	85.8	85.8	85.8	85.8	85.8	85.8	57.2	57.2	57.2
**14**	74.9	74.9	74.9	74.9	74.9	74.9	74.9	74.9	74.9	74.9	49.9	49.9
**15**	63.7	63.7	63.7	63.7	63.7	63.7	63.7	63.7	63.7	63.7	63.7	42.5
**16**	52.7	52.7	52.7	52.7	52.7	52.7	52.7	52.7	52.7	52.7	52.7	52.7
**17**	42.3	42.3	42.3	42.3	42.3	42.3	42.3	42.3	42.3	42.3	42.3	42.3
**18**	32.8	32.8	32.8	32.8	32.8	32.8	32.8	32.8	32.8	32.8	32.8	32.8
**19**	24.6	24.6	24.6	24.6	24.6	24.6	24.6	24.6	24.6	24.6	24.6	24.6
**20**	17.7	17.7	17.7	17.7	17.7	17.7	17.7	17.7	17.7	17.7	17.7	17.7
**21**	12.0	12.0	12.0	12.0	12.0	12.0	12.0	12.0	12.0	12.0	12.0	12.0
**22**	7.3	7.3	7.3	7.3	7.3	7.3	7.3	7.3	7.3	7.3	7.3	7.3
**23**	3.8	3.8	3.8	3.8	3.8	3.8	3.8	3.8	3.8	3.8	3.8	3.8
**24**	1.5	1.5	1.5	1.5	1.5	1.5	1.5	1.5	1.5	1.5	1.5	1.5
**25**	0.4	0.4	0.4	0.4	0.4	0.4	0.4	0.4	0.4	0.4	0.4	0.4
**26**	0.1	0.1	0.1	0.1	0.1	0.1	0.1	0.1	0.1	0.1	0.1	0.1
**27**	0.0	0.0	0.0	0.0	0.0	0.0	0.0	0.0	0.0	0.0	0.0	0.0
**28**	0.0	0.0	0.0	0.0	0.0	0.0	0.0	0.0	0.0	0.0	0.0	0.0
**29**	0.0	0.0	0.0	0.0	0.0	0.0	0.0	0.0	0.0	0.0	0.0	0.0
**30**	0.0	0.0	0.0	0.0	0.0	0.0	0.0	0.0	0.0	0.0	0.0	0.0
**All Age 1+ F**	**1841**	**1809**	**1863**	**1953**	**2000**	**2041**	**2081**	**2116**	**2196**	**2243**	**2331**	**2382**
**All Age1+ MF**	**2615**	**2580**	**2718**	**2884**	**2963**	**3029**	**3098**	**3158**	**3309**	**3381**	**3538**	**3608**

**Fig 3:**
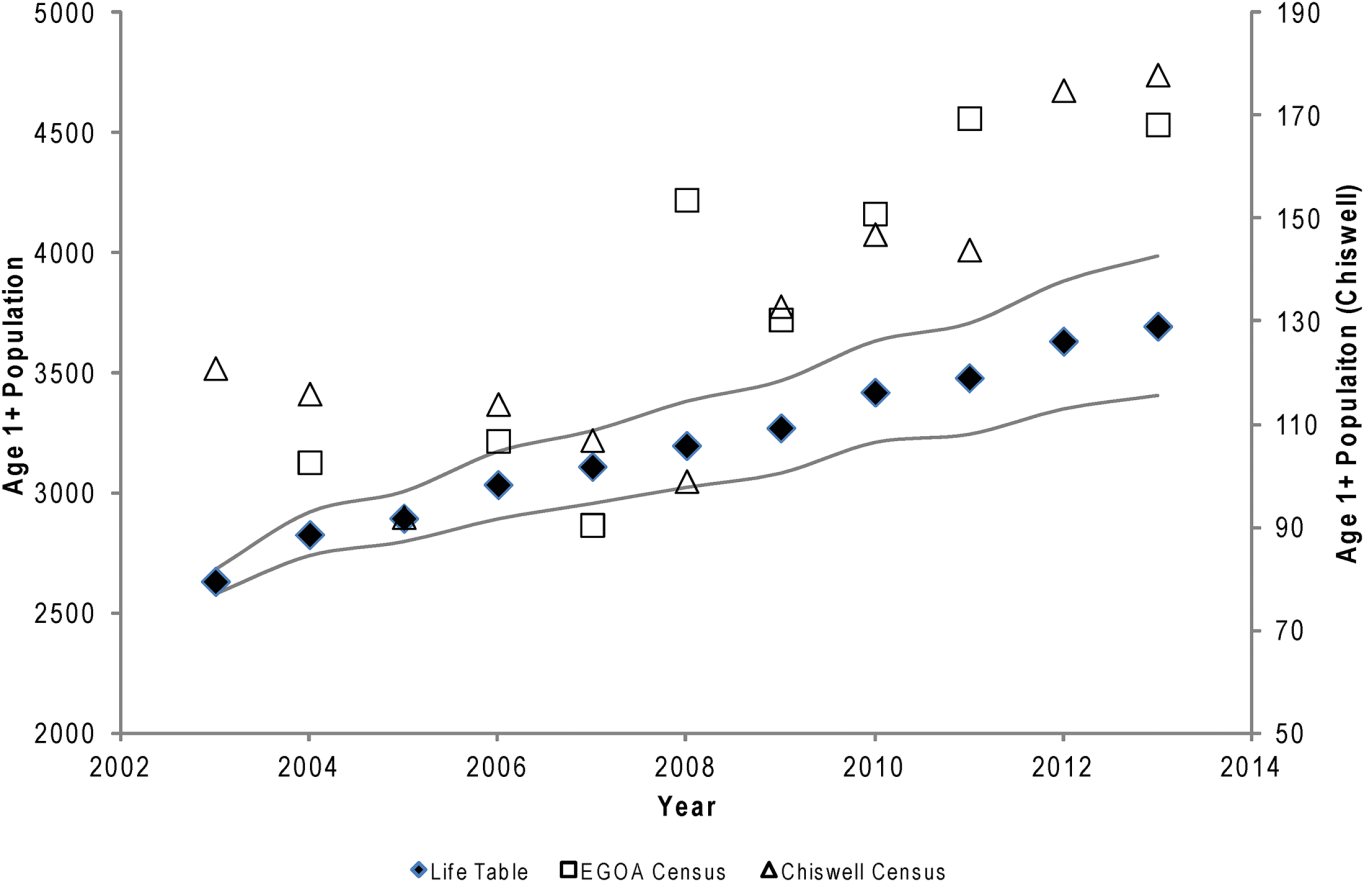
Population trend for Steller sea lions estimated from life tables (Table 4.2; black diamonds ±95% CI: r^2^ = 0.987, *P* < 0.001), census counts across the eastern Gulf of Alaska (open squares), and Chiswell Island census counts (open triangles).

The reconstructed population of age 1+ Steller sea lions increased at a mean rate of 3.5% per year (Fig 3). These population estimates were correlated with census counts of the breeding and juvenile population at Chiswell Island between 2003 and 2013 (r^2^ = 0.622, *P* = 0.004), and at index sites throughout the entire EGOA between 2004 and 2011 (r^2^ = 0.688, *P* = 0.011). The population at Chiswell Island and in the EGOA appears to increase more strongly after 2007 than predicted by the population model (Fig 3).

**Fig 4:**
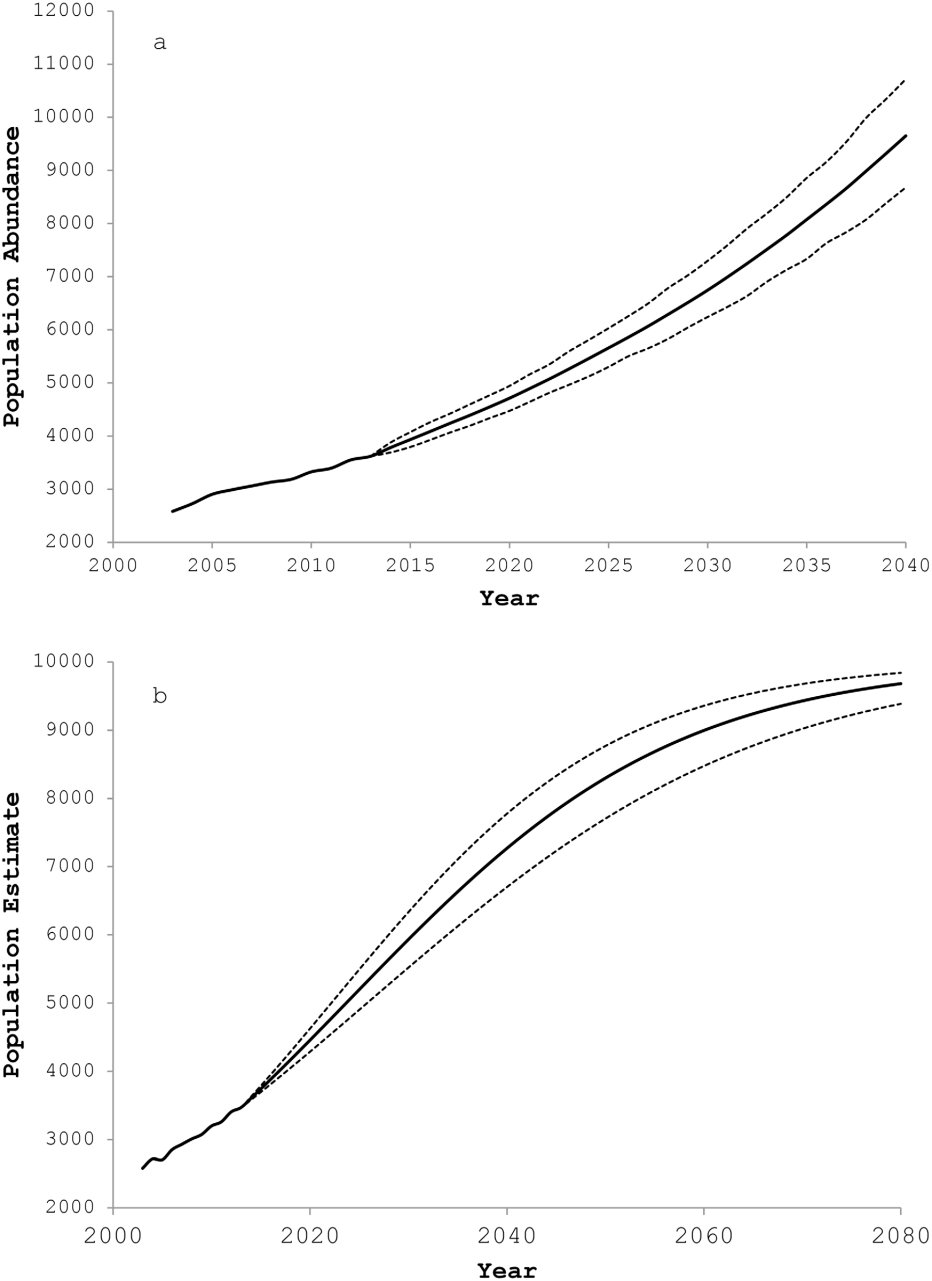
(a) Density-independent projections (95% C.I.) of the Steller sea lion population in the eastern Gulf of Alaska based on bootstrapped values of fecundity and survival observed over the years 2003–2012. (b) Projected population trend (95% C.I.) for Steller sea lions in the eastern Gulf of Alaska based on an intrinsic rate of increase of *r* = 0.059 and carrying capacity of *K* = 10,000 age 1+ males and females.

Projecting the population into the future based on the observed variation in fecundity and survival and without regard to density dependence resulted in rapidly increasing numbers (Fig 4a). With this scenario, we could expect the EGOA population to reach 90% of carrying capacity (*K*) by 2038 (95% C.I.: 2036–2041). The maximum instantaneous rate of increase (*r*) over the study period was 0.059. Considering this rate, and with *K* set at 10,000 individuals, the population in the EGOA should reach 90% of *K* in the year 2060 (95% C.I.: 2053–2070; Fig 4b).

**Fig 5:**
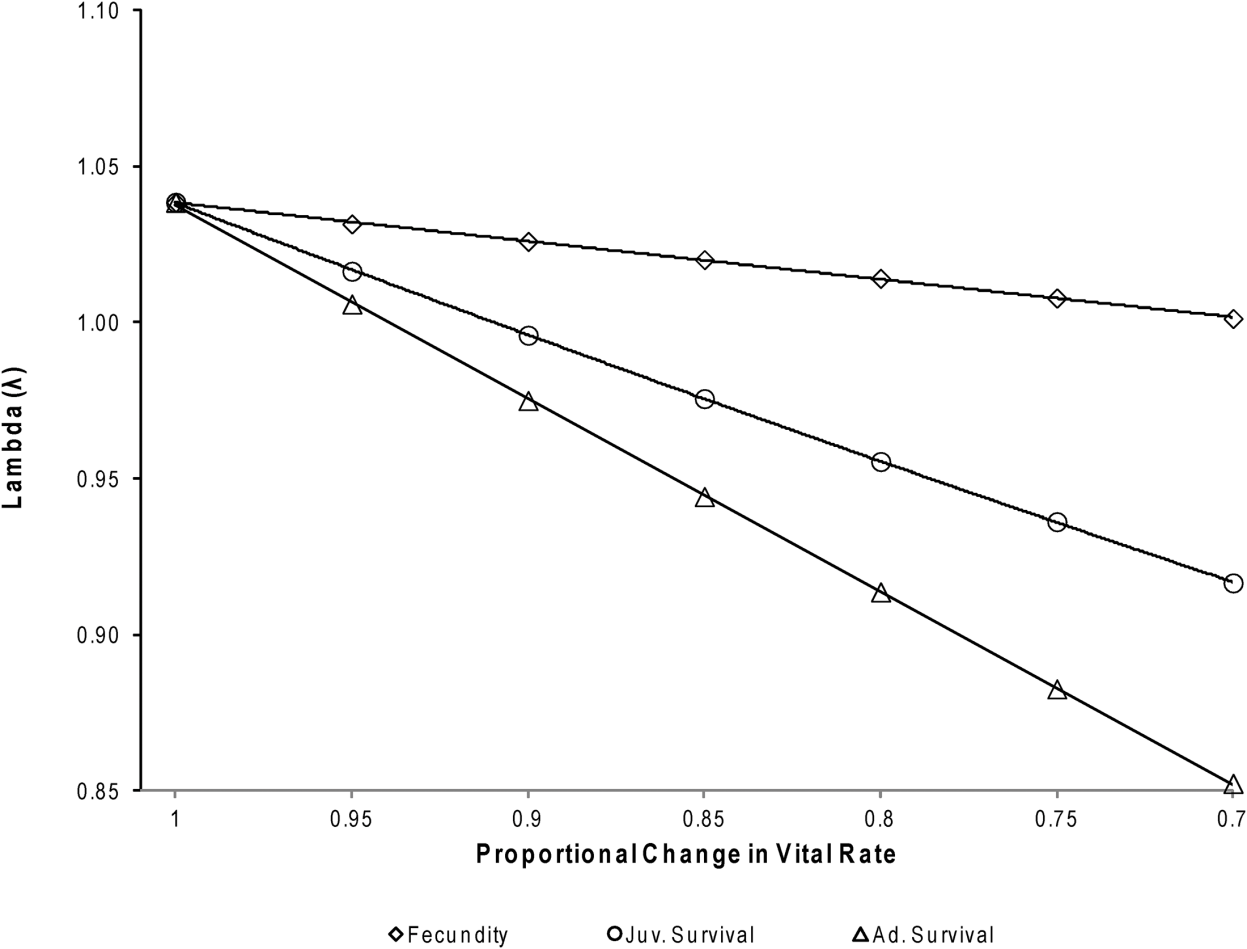
Expected change in λ based on proportional changes to individual vital rates (λ less than 1 indicates a declining population).

**Fig 6:**
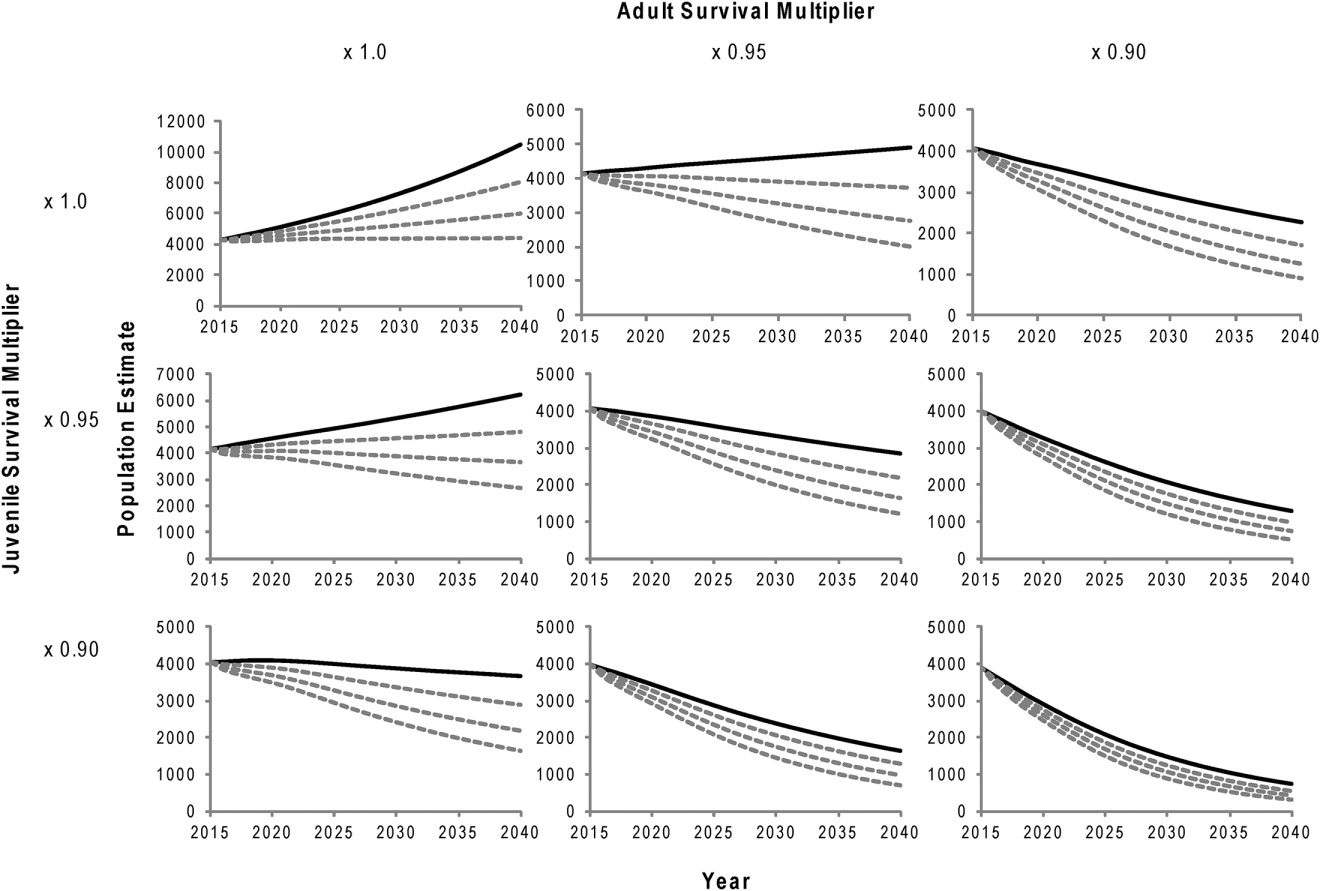
Population projections to 2040 based on multiple changes in vital rates. Left to right represents reductions in adult survival from 100% to 95% to 90%, whereas top to bottom represents reductions in juvenile survival from 100% to 95% to 90%, of current estimates. The dark line within each graph represents no change in current rates of fecundity, whereas the lower 3 dotted lines represent change in fecundity to 90%, 80%, and 70% in decreasing fashion. Note different scales in the y-axes.

By proportionally varying vital rates in a prospective elasticity analysis, we found that fecundity had very little effect on λ, whereas juvenile survival and adult survival had much larger impacts (Fig 5). A population decline (λ < 1.0) could be expected with only a 6% decrease in adult survival or a 9% decrease in juvenile survival. On the other hand, it would take a 32% decrease in fecundity for λ to drop below 1.0. Average age of female maturity (age at first pupping as presented here) would also have to change from 5 years to about 10 years of age for the population forecast to decline. Since vital rates likely do not vary independently of each other [2,30], several scenarios are presented in Fig 6, expressing the predicted responses to multiple insults to the primary vital rates presented in this work.

Finally, modeling negative covariation between fecundity and juvenile survival results in roughly a 1.9% increase in survival to age 2 for females and 3.8% increase for males with every 10% reduction in fecundity, if the same proportion of females not giving birth continue to suckle their pups for an additional year. Opposing changes in fecundity and second year survival would mean that a reduction in fecundity to 57% of the currently observed rates would be required over the next 25 years to cause a decline in Steller sea lion populations.

## Discussion

Several otariid (sea lions and fur seals) populations have fully recovered from collapses in abundance that have been severe enough to be termed catastrophic [26]. The reasons for those catastrophes varied from anthropogenic effects of fisheries or directed harvest to natural environmental influences such as El Niño events or disease agents (summarized in [26]). Steller sea lions in Alaska may have declined in numbers for a variety of reasons [21,23,45,46], but regardless of the cause(s), they are now emulating the trends of other otariids in their ability to recover.

The upsurge in numbers is especially strong in the eastern Gulf of Alaska [24] and may be fueled by good juvenile survival [14,16] and good reproductive rates [17]. The correlations between the population trend modeled here and actual population counts suggests that monitoring vital rates at a local level can provide a good indication of broader population dynamics for Steller sea lions. The rate of increase estimated here is highly consistent with broad-scale estimates based on population counts across much of the Gulf of Alaska and Eastern Aleutian Islands over the past decade [24]. However, the count data presented in Fig 2 indicate that there may have been a more rapid increase in numbers beginning around 2008 in the EGOA than indicated by the modeled population trend. There could be a number of reasons for this apparent upsurge that are discussed as follows, but the most likely contributing factor is immigration.

From a sampling perspective, there can be a degree of uncertainty with population counts conducted once or twice per year, and not in every year, due to process noise and observation error [47]. Among pinnipeds, uncertainties exist primarily due to process noise, mostly in the form of an unknown proportion of animals being hauled out during the time of the survey count because of a variety of environmental influences [48,49]. One potential influence is an apparent ecosystem change beginning in 2008 related to the Pacific Decadal Oscillation that stimulated greater production of energy-rich fish such as capelin (*Mallotus villosus*) in the study area [50]. This change could have caused a positive trend in the numbers of animals hauled out and available for observation as animals that find abundant food resources nearby their rookeries may spend less time foraging at sea as explained by Maniscalco et al. [25]. The increase in abundance of fishes such as capelin may have also had positive influences on reproduction and survival since 2008, although little change was detected in Steller sea lion vital rates over the 2003–2013 study period [16,17]. Another possible explanation for the recent upsurge in numbers is that there may have been a change in first-year survival between the early 2000s and the late 2000s from 55% [14] to 80% [16]. Coincidentally, there was a relaxation of killer whale predation on young Steller sea lions at the Chiswell Island rookery after 2004 [51]. However, this change in first year survival was taken into account in this analysis and could not explain the rapid increase in numbers in the most recent years.

Based on branded animal sightings throughout Alaska, Fritz et al. [33] estimate about 1700 Steller sea lions immigrated to the EGOA between 2000 and 2011, most of them from the central Gulf. This figure largely explains the discrepancy between the population trend modeled here and survey counts in the EGOA. Many of these animals were juveniles and may be temporary migrants, but some have also remained and joined the breeding population [33] further contributing to the EGOA surge in numbers.

Regardless of the ultimate cause(s), the rapid increase of Steller sea lion numbers in the EGOA suggests that there is little density-dependent control on the population at present. If current trends and density independence are to be expected into the near future, this region of the WDPS should return to peak numbers within about 23 years (Fig 4a). Immigration will result in an even quicker return to full recovery in this sub-region [33]. Should density dependent factors come into play, the recovery may take twice as long according to population models presented here. The current rate of increase is close to that seen in other pinniped populations recovering from drastic declines, such as northern elephant seals (*Mirounga angustirostris*) [52], gray seals (*Halichoerus grypus*) [42], South American sea lions (*Otaria flavescens*) [53], and New Zealand fur seals (*Arctocephalus forsteri*) [54]. Those populations have been increasing by 4–13% per year in recent decades and some have experienced an exponential growth phase [42], similar to what we may now, or may soon be, observing with Steller sea lions in the EGOA.

Projecting the population into the future, we found that trends showed the least elasticity to variation in fecundity and were most elastic in regards to variation in adult survival, as would be expected for a long-lived *K*-selected species [55]. In mammals that have delayed maturity and low reproductive rates, survival is much more influential on population growth than fecundity [55,56]. Population growth in the presence of environmental stochasticity is typically buffered in long-lived mammals by stability in adult survival, followed by juvenile survival and fecundity [2,30]. York [8] suggested that a 10–20% decrease in juvenile survival was the predominant driver of the population decline between the 1970s and 1980s with an insignificant change in adult survival. This is consistent with the demographic buffering hypothesis [30,31] in which significant decreases in adult survival among long lived species are rarely seen because this would have greater detrimental effects on the population. Among pinnipeds, this type of demographic buffering has also been found in Weddell seals (*Leptonychotes weddelli*) [32]. However, in Steller sea lions, variation in juvenile survival may also be strongly affected by the duration of maternal care [16].

Similar to variation in fecundity, variation in the age at first reproduction did not greatly influence population trends, as an increase from 5 to 10 years was needed to observe a negative population trend. Age at pregnancy for Steller sea lions did not contribute to model fit when comparing fecundity rates between the 1970s and 1980s [41]. Therefore, it is unlikely that a change in average age at maturity occurred between those time periods, or if it had, it would have been minimal and unlikely to significantly impact population trends.

It is also suggested that Steller sea lions may have an additional buffering mechanism in which a reduction in fecundity could result in an increase in juvenile survival between ages 1 and 2 [16]. In many cases, vital rates will vary in the same direction [29,32], as they apparently did for Steller sea lions during the period of the decline when fecundity declined along with juvenile survival [8]. Yet, whether vital rates covary negatively or positively will depend on the source of environmental pressures [57]. Negatively varying vital rates are usually observed as a cost of reproduction [58], where females that reproduce often have reduced chances of survival. Depending on environmental conditions and life history characteristics, females of some avian and mammalian species may forego their own fitness in favor of enhancing the survival likelihood of their offspring [59]. However, under some circumstances, a different pattern of life history variation may occur in Steller sea lions, and perhaps other long-lived mammals that have large variations in the duration of maternal care. That is, mature females that experience a modest reduction in food availability or increase in disease may forego reproduction or abort their fetus [41]. These females that skip a year of giving birth, commonly continue to nurse their dependent offspring for an additional year [19] and such prolonged maternal care has been shown to greatly improve juvenile survival [16]. This life-history strategy makes sense from an evolutionary perspective, because juvenile survival has a much greater impact on population health compared to reproduction as shown here. Therefore, when times are tough, juveniles can still maintain good survivorship if their mothers forego pregnancy. However, as mentioned, the population decline was caused primarily by a reduction in juvenile survival, concurrent with minor reductions in fecundity [8].

The evidence for effects of dietary changes on the health and survival of juvenile and adult sea lions is sparse and highly equivocal [60–62]. The effect of diet on fecundity is also unknown for Steller sea lions, but a change in the population trend is unlikely unless fecundity dropped by more than 30% for a prolonged period, and there is no evidence for that [17,25]. On the other hand, killer whale predation [63,64] and anthropogenic effects [21,65] can affect survival of all age classes. An estimated 20,000 Steller sea lions were killed by direct interactions with the fishing industry (primarily shooting and net entanglements) between 1968 and 1985, and most were females [22,65]. Fisheries restrictions around major rookeries apparently had a mitigating effect on the population decline [66], but regardless, those losses alone cannot explain the population crash. Additionally, a small number of killer whales focusing their predation on Steller sea lions could effectively collapse the population [64]. These studies suggest that the major declines in Steller sea lion numbers between the 1970s and 2000 may have been multifactorial, but driven primarily by predation and/or direct effects of commercial fisheries (shooting and entanglement), because a drastic population decline in Steller sea lions cannot be explained by moderate reductions in fecundity. Yet, a moderate decrease in juvenile survival or a modest decline in adult survival can cause severe population declines as modeled here.

Much research effort has been dedicated to studying reproduction in Steller sea lions since a theoretical population model suggested declines in natality were occurring well into the 2000s, and were a cause for continued concern [10]. Aside from the untenable assumptions found in that work [25], empirical studies have shown natality rates for Steller sea lions have been relatively high in the Gulf of Alaska during the 2000s [17,25]. Furthermore, there has been little consideration of the elasticities of fecundity and other vital rates in recent years. For example, survival rates of Steller sea lions in the Russian Far East were recently found to be low at Medny Island compared to other areas, as much as 8% lower for adults [13]. However, the authors discount the meaning of this finding in favor of future investigations of reproductive rates. It is likely, however, that they have found the proximate mechanism for the decline at Medny Island in reduced survival rates. Naturally, investigations of fecundity and reproductive biology should be undertaken, as they represent an important vital rate and will continue to be topics of interest for a wide variety of biologists. Yet, to identify causes for the Steller sea lion population decline or its failure to recover, we need to understand the relative effects of these vital rates on population trends. Then we can prioritize investigations of ecological variables that will have the greatest influence on the vital rates with the highest elasticities.

The recovery of much of the western population of Steller sea lions is well underway [24], but looking to the future, we can expect the greatest threats will be factors that have an effect on survival of adults and juveniles. Minor changes in diet are unlikely to have substantial impacts on adult and juvenile survival, as Steller sea lions are adaptable to a varied diet [60,61]. Mortality based on conflicts with fisheries has been mitigated due to laws, fishing restrictions, and greater awareness and, therefore, not expected to be a future threat [21]. Predation, primarily by killer whales, but also by sleeper sharks (*Somniosus pacificus*), does remain as a potential threat to recovery [64,67,68] and should continue to be monitored. Changes in the population trend of Steller sea lions can be detected by monitoring vital rates at a representative rookery using a variety of mark-recapture statistics. Furthermore, specific threats to the population can be better pinpointed with such detailed monitoring in contrast to survey counts alone. The Chiswell Island remote video monitoring project [25,37] is an excellent study site for continued monitoring of Steller sea lion vital rates and a variety of other behavioral and population-based research objectives.

## Supporting Information

S1 Table Complete life table survival parameters used for modeling the Steller sea lion female (a) and male (b) proportions of the population in the Eastern Gulf of Alaska. (DOCX)
