# Supplementary material for: Population Trend and Elasticities of Vital Rates for Steller Sea Lions (Eumetopias jubatus) in the Eastern Gulf of Alaska: A New Life-History Table Analysis
Source: PLoS One. 2015 Oct 21;10(10):e0140982. doi: 10.1371/journal.pone.0140982 (PMC4619567; doi:10.1371/journal.pone.0140982)
Supplement: S1 Table — (DOCX) [file pone.0140982.s001.docx]

Appendix 1: Complete life table survival parameters used for modeling the Steller sea lion female (a) and male (b) proportions of the population in the Eastern Gulf of Alaska.

a)

| **Year** | **2003** | **2004** | **2005** | **2006** | **2007** | **2008** | **2009** | **2010** | **2011** | **2012** | **2013** |
| --- | --- | --- | --- | --- | --- | --- | --- | --- | --- | --- | --- |
| **to Age 1** | 0.508 | 0.534 | 0.712 | 0.736 | 0.721 | 0.717 | 0.710 | 0.765 | 0.705 | 0.729 | 0.678 |
| **2** | 0.696 | 0.696 | 0.696 | 0.696 | 0.696 | 0.696 | 0.696 | 0.696 | 0.696 | 0.696 | 0.696 |
| **3** | 0.921 | 0.921 | 0.921 | 0.921 | 0.921 | 0.921 | 0.921 | 0.921 | 0.921 | 0.921 | 0.921 |
| **4** | 0.941 | 0.941 | 0.941 | 0.941 | 0.941 | 0.941 | 0.941 | 0.941 | 0.941 | 0.941 | 0.941 |
| **5** | 0.958 | 0.958 | 0.958 | 0.958 | 0.958 | 0.958 | 0.958 | 0.958 | 0.958 | 0.958 | 0.958 |
| **6** | 0.968 | 0.968 | 0.968 | 0.968 | 0.968 | 0.968 | 0.968 | 0.968 | 0.968 | 0.968 | 0.968 |
| **7** | 0.969 | 0.969 | 0.969 | 0.969 | 0.969 | 0.969 | 0.969 | 0.969 | 0.969 | 0.969 | 0.969 |
| **8** | 0.970 | 0.970 | 0.970 | 0.970 | 0.970 | 0.970 | 0.970 | 0.970 | 0.970 | 0.970 | 0.970 |
| **9** | 0.959 | 0.959 | 0.959 | 0.959 | 0.959 | 0.959 | 0.959 | 0.959 | 0.959 | 0.959 | 0.959 |
| **10** | 0.945 | 0.945 | 0.945 | 0.945 | 0.945 | 0.945 | 0.945 | 0.945 | 0.945 | 0.945 | 0.945 |
| **11** | 0.930 | 0.930 | 0.930 | 0.930 | 0.930 | 0.930 | 0.930 | 0.930 | 0.930 | 0.930 | 0.930 |
| **12** | 0.913 | 0.913 | 0.913 | 0.913 | 0.913 | 0.913 | 0.913 | 0.913 | 0.913 | 0.913 | 0.913 |
| **13** | 0.894 | 0.894 | 0.894 | 0.894 | 0.894 | 0.894 | 0.894 | 0.894 | 0.894 | 0.894 | 0.894 |
| **14** | 0.873 | 0.873 | 0.873 | 0.873 | 0.873 | 0.873 | 0.873 | 0.873 | 0.873 | 0.873 | 0.873 |
| **15** | 0.851 | 0.851 | 0.851 | 0.851 | 0.851 | 0.851 | 0.851 | 0.851 | 0.851 | 0.851 | 0.851 |
| **16** | 0.827 | 0.827 | 0.827 | 0.827 | 0.827 | 0.827 | 0.827 | 0.827 | 0.827 | 0.827 | 0.827 |
| **17** | 0.802 | 0.802 | 0.802 | 0.802 | 0.802 | 0.802 | 0.802 | 0.802 | 0.802 | 0.802 | 0.802 |
| **18** | 0.776 | 0.776 | 0.776 | 0.776 | 0.776 | 0.776 | 0.776 | 0.776 | 0.776 | 0.776 | 0.776 |
| **19** | 0.749 | 0.749 | 0.749 | 0.749 | 0.749 | 0.749 | 0.749 | 0.749 | 0.749 | 0.749 | 0.749 |
| **20** | 0.722 | 0.722 | 0.722 | 0.722 | 0.722 | 0.722 | 0.722 | 0.722 | 0.722 | 0.722 | 0.722 |
| **21** | 0.676 | 0.676 | 0.676 | 0.676 | 0.676 | 0.676 | 0.676 | 0.676 | 0.676 | 0.676 | 0.676 |
| **22** | 0.609 | 0.609 | 0.609 | 0.609 | 0.609 | 0.609 | 0.609 | 0.609 | 0.609 | 0.609 | 0.609 |
| **23** | 0.518 | 0.518 | 0.518 | 0.518 | 0.518 | 0.518 | 0.518 | 0.518 | 0.518 | 0.518 | 0.518 |
| **24** | 0.403 | 0.403 | 0.403 | 0.403 | 0.403 | 0.403 | 0.403 | 0.403 | 0.403 | 0.403 | 0.403 |
| **25** | 0.273 | 0.273 | 0.273 | 0.273 | 0.273 | 0.273 | 0.273 | 0.273 | 0.273 | 0.273 | 0.273 |
| **26** | 0.148 | 0.148 | 0.148 | 0.148 | 0.148 | 0.148 | 0.148 | 0.148 | 0.148 | 0.148 | 0.148 |
| **27** | 0.054 | 0.054 | 0.054 | 0.054 | 0.054 | 0.054 | 0.054 | 0.054 | 0.054 | 0.054 | 0.054 |
| **28** | 0.008 | 0.008 | 0.008 | 0.008 | 0.008 | 0.008 | 0.008 | 0.008 | 0.008 | 0.008 | 0.008 |
| **29** | 0.000 | 0.000 | 0.000 | 0.000 | 0.000 | 0.000 | 0.000 | 0.000 | 0.000 | 0.000 | 0.000 |
| **30** | 0.000 | 0.000 | 0.000 | 0.000 | 0.000 | 0.000 | 0.000 | 0.000 | 0.000 | 0.000 | 0.000 |

b)

| **Year** | **2003** | **2004** | **2005** | **2006** | **2007** | **2008** | **2009** | **2010** | **2011** | **2012** | **2013** |
| --- | --- | --- | --- | --- | --- | --- | --- | --- | --- | --- | --- |
| **to Age 1** | 0.549 | 0.578 | 0.712 | 0.736 | 0.721 | 0.717 | 0.710 | 0.765 | 0.705 | 0.729 | 0.678 |
| **2** | 0.486 | 0.486 | 0.486 | 0.486 | 0.486 | 0.486 | 0.486 | 0.486 | 0.486 | 0.486 | 0.486 |
| **3** | 0.821 | 0.821 | 0.821 | 0.821 | 0.821 | 0.821 | 0.821 | 0.821 | 0.821 | 0.821 | 0.821 |
| **4** | 0.821 | 0.821 | 0.821 | 0.821 | 0.821 | 0.821 | 0.821 | 0.821 | 0.821 | 0.821 | 0.821 |
| **5** | 0.930 | 0.930 | 0.930 | 0.930 | 0.930 | 0.930 | 0.930 | 0.930 | 0.930 | 0.930 | 0.930 |
| **6** | 0.930 | 0.930 | 0.930 | 0.930 | 0.930 | 0.930 | 0.930 | 0.930 | 0.930 | 0.930 | 0.930 |
| **7** | 0.921 | 0.921 | 0.921 | 0.921 | 0.921 | 0.921 | 0.921 | 0.921 | 0.921 | 0.921 | 0.921 |
| **8** | 0.912 | 0.912 | 0.912 | 0.912 | 0.912 | 0.912 | 0.912 | 0.912 | 0.912 | 0.912 | 0.912 |
| **9** | 0.892 | 0.892 | 0.892 | 0.892 | 0.892 | 0.892 | 0.892 | 0.892 | 0.892 | 0.892 | 0.892 |
| **10** | 0.860 | 0.860 | 0.860 | 0.860 | 0.860 | 0.860 | 0.860 | 0.860 | 0.860 | 0.860 | 0.860 |
| **11** | 0.809 | 0.809 | 0.809 | 0.809 | 0.809 | 0.809 | 0.809 | 0.809 | 0.809 | 0.809 | 0.809 |
| **12** | 0.739 | 0.739 | 0.739 | 0.739 | 0.739 | 0.739 | 0.739 | 0.739 | 0.739 | 0.739 | 0.739 |
| **13** | 0.652 | 0.652 | 0.652 | 0.652 | 0.652 | 0.652 | 0.652 | 0.652 | 0.652 | 0.652 | 0.652 |
| **14** | 0.550 | 0.550 | 0.550 | 0.550 | 0.550 | 0.550 | 0.550 | 0.550 | 0.550 | 0.550 | 0.550 |
| **15** | 0.434 | 0.434 | 0.434 | 0.434 | 0.434 | 0.434 | 0.434 | 0.434 | 0.434 | 0.434 | 0.434 |
| **16** | 0.306 | 0.306 | 0.306 | 0.306 | 0.306 | 0.306 | 0.306 | 0.306 | 0.306 | 0.306 | 0.306 |
| **17** | 0.168 | 0.168 | 0.168 | 0.168 | 0.168 | 0.168 | 0.168 | 0.168 | 0.168 | 0.168 | 0.168 |
| **18** | 0.023 | 0.023 | 0.023 | 0.023 | 0.023 | 0.023 | 0.023 | 0.023 | 0.023 | 0.023 | 0.023 |
| **19** | 0.001 | 0.001 | 0.001 | 0.001 | 0.001 | 0.001 | 0.001 | 0.001 | 0.001 | 0.001 | 0.001 |
| **20** | 0.001 | 0.001 | 0.001 | 0.001 | 0.001 | 0.001 | 0.001 | 0.001 | 0.001 | 0.001 | 0.001 |
| **21** | 0.001 | 0.001 | 0.001 | 0.001 | 0.001 | 0.001 | 0.001 | 0.001 | 0.001 | 0.001 | 0.001 |
| **22** | 0.001 | 0.001 | 0.001 | 0.001 | 0.001 | 0.001 | 0.001 | 0.001 | 0.001 | 0.001 | 0.001 |
| **23** | 0.001 | 0.001 | 0.001 | 0.001 | 0.001 | 0.001 | 0.001 | 0.001 | 0.001 | 0.001 | 0.001 |
| **24** | 0.000 | 0.000 | 0.000 | 0.000 | 0.000 | 0.000 | 0.000 | 0.000 | 0.000 | 0.000 | 0.000 |
| **25** | 0.000 | 0.000 | 0.000 | 0.000 | 0.000 | 0.000 | 0.000 | 0.000 | 0.000 | 0.000 | 0.000 |
| **26** | 0.000 | 0.000 | 0.000 | 0.000 | 0.000 | 0.000 | 0.000 | 0.000 | 0.000 | 0.000 | 0.000 |
| **27** | 0.000 | 0.000 | 0.000 | 0.000 | 0.000 | 0.000 | 0.000 | 0.000 | 0.000 | 0.000 | 0.000 |
| **28** | 0.000 | 0.000 | 0.000 | 0.000 | 0.000 | 0.000 | 0.000 | 0.000 | 0.000 | 0.000 | 0.000 |
| **29** | 0.000 | 0.000 | 0.000 | 0.000 | 0.000 | 0.000 | 0.000 | 0.000 | 0.000 | 0.000 | 0.000 |
| **30** | 0.000 | 0.000 | 0.000 | 0.000 | 0.000 | 0.000 | 0.000 | 0.000 | 0.000 | 0.000 | 0.000 |
